# Ketogenic Effects of Multiple Doses of a Medium Chain Triglycerides Enriched Ketogenic Formula in Healthy Men under the Ketogenic Diet: A Randomized, Double-Blinded, Placebo-Controlled Study

**DOI:** 10.3390/nu14061199

**Published:** 2022-03-12

**Authors:** Kentaro Nakamura, Keisuke Hagihara, Naoko Nagai, Ryuichiro Egashira, Mariko Takeuchi, Mai Nakano, Hitomi Saito, Misaki Moriguchi, Satoko Tonari, Satoshi Watanabe, Akimitsu Miyake, Kinya Ashida

**Affiliations:** 1 Research Team 3, Co-Creation Center, Meiji Holdings Co., Ltd., Tokyo 192-0919, Japan; kentarou.nakamura@meiji.com (K.N.); kinya.ashida@meiji.com (K.A.); 2 Department of Advanced Hybrid Medicine, Graduate School of Medicine, Osaka University, Osaka 565-0871, Japan; r_egashira@kanpou.med.osaka-u.ac.jp (R.E.); m_takeuchi@kanpou.med.osaka-u.ac.jp (M.T.); norse.vsta.m@gmail.com (M.N.); hitomis.og@gmail.com (H.S.); moriguchi@kanpou.med.osaka-u.ac.jp (M.M.); tonari@kanpou.med.osaka-u.ac.jp (S.T.); 3 Division of Nutritional Management, Osaka University Hospital, Osaka 565-0871, Japan; nagaink@hosp.med.osaka-u.ac.jp; 4 Cykinso, Inc., Tokyo 151-0053, Japan; s.watanabe@cykinso.co.jp; 5 Department of Medical Innovation, Osaka University Hospital, Osaka 565-0871, Japan; akimitsu.miyake@dmi.med.osaka-u.ac.jp

**Keywords:** ketogenic diet, ketogenic formula, multiple dose study, ketone bodies, gut microbiota

## Abstract

Ketogenic diets, which are carbohydrate-restricted high-fat diets, may have therapeutic effects on various diseases, including cancer. However, ketogenic diets are often not standardized and, therefore, results are difficult to interpret. We previously investigated the usefulness of ketogenic diets in cancer therapy, where ketogenic formulas (KF) were used as supplements to enhance blood ketone bodies; however, the amount of KF was determined empirically with reference to blood ketone bodies levels. Here, to determine a standardized optimal amount of KF, we investigated temporal changes in blood ketone bodies (acetoacetic acid (AcAc), β-hydroxybutyrate (BHB)) and safety in 20 healthy individuals when KF was taken repeatedly under the conditions of a ketogenic diet (UMIN000034216). The diurnal variation in total ketone bodies, and AcAc and BHB levels significantly increased after lunch and after dinner, on the 4th day of KF administration. There were no significant safety issues related to KF in the context of anthropometric, metabolic, nutritional, urological and gastrointestinal parameters. In addition, ketogenic diets lead to changes in gut microbiota. KF showed a decrease in phylum *Firmicutes*. Our study provides baseline data of the usefulness of KF in a ketogenic diet.

## 1. Introduction

Due to the advances in diagnostic imaging, malignancies, such as gastric cancer and colorectal cancer, can be detected early and resected, which leads to improvements in patient prognoses. However, for malignancies that are detected as advanced cancers, such as pancreatic cancer, gallbladder cancer, and lung cancer, prognoses have obviously not improved [1]. Fat-rich diets are one of the greatest risk factors for the development of colorectal cancer and breast cancer; however, it has been reported that fat intake is not a risk for developing colorectal cancer and breast cancer in an eight-year follow-up survey of about 50,000 menopausal women in the United States [2]. Prior to 1910, Inuit people had lower blood lipid levels and a much lower incidence of cancer than Danish people in all age groups, even though they maintained a traditional diet similar to the ketogenic diet (low-carbohydrate and high-fat diet) and consumed fat in excess of 40% of total energy intake [3]. However, Western food culture has been adopted by Inuit people since 1910, and the incidence of cancers, which have been observed in Western countries, such as colorectal cancer, lung cancer, breast cancer, and prostate cancer, have increased sharply as of the 1950s [4]. In Japan, there has also been an increase in cancers that are often found in Europe and the United States, such as colon cancer, breast cancer, and lung cancer, because of the westernization of the Japanese diet. Men with high C peptide levels, compared to men with low C peptide levels, are up to three times more likely to develop colorectal cancer [5].

The ketogenic diet has a long history. In Ancient Greece, Hippocrates reported the effects of fasting on epilepsy, and Wilder devised the ketogenic diet in 1921 as one that is less burdensome to the body than fasting [6]. The efficacy and safety of the ketogenic diet for patients with refractory epilepsy (Glut-1 deficiency) have been confirmed [7]. Recently, the relationship between carbohydrates and carcinogenesis has also been a subject of focus, and the ketogenic diet is expected to have therapeutic effects in cancer patients. In vivo, 50% of HER-2/neu-expressing mice in a normal diet group developed breast cancer in one year, while no carcinogenesis was observed in a low-carbohydrate, high-protein diet group [8]. In clinical, the beneficial effects of the ketogenic diet has been reported in a 65-year-old female glioblastoma patient, who was also taking anticancer drugs and undergoing radiation therapy, who experienced rapid regression [9]. In Germany, 5 of 16 terminal cancer patients who received a low-carbohydrate diet prolonged their survival and improved their quality of life (QOL) in the contexts of insomnia, mental anxiety, and loss of appetite [10]. However, there has been no clear evidence of the beneficial effects of a ketogenic diet in cancer patients in Japan. We recently reported case studies of patients with various carcinomas (stage IV) and showed that the ketogenic diet may be a promising supportive care option for patients with various types of advanced cancer [11]; however, in order to build a foundation of more robust evidence on the usefulness of the ketogenic diet for cancer in clinical trials, it is essential to standardize the ketogenic diet regimen for cancer patients. In our previous study, we implemented a ketogenic diet for cancer based on the clinical experience of ketogenic diets for patients with refractory epilepsy [11]. In this ketogenic diet therapy, medium-chain triglycerides (MCT) and ketogenic formulas (KF) were appropriately used as supplements for enhancing blood ketone bodies [11]. However, the amount of KF used was determined based on practical experiences, while referring to blood ketone body levels. Therefore, to determine the optimal amount of KF to be used, and to standardize the ketogenic diet regimen, we investigated changes in blood ketone bodies (acetoacetic acid and β-hydroxybutyrate) and the safety of healthy individuals when KF was taken repeatedly under the conditions of a ketogenic diet.

## 2. Materials and Methods

### 2.1. Subjects

Written informed consent was obtained from all participants. The inclusion criteria were as follows: healthy male adult, aged 20 to 40; BMI of 18.5 to 25.0; height of 165 cm to 175 cm. The exclusion criteria were as follows: smoking habits; receiving drug treatments; blood test values that deviate significantly from standard values; digestive disorders; a history of food allergies; lactose intolerance; already on a carbohydrate-restricted diet or ketogenic diet; and subjects who were judged inappropriate for the study by the principal investigator due to abnormal blood parameters or other reasons. This study was conducted with the approval of the ethics review committee of Osaka University and Meiji Co., Ltd. (approval number: 18191 and 2018-015, respectively), and was registered in the University Hospital Information Network (UMIN) clinical trial system before the enrollment of subjects (UMIN000034216).

### 2.2. Case Registration and Randomization

Written consent was obtained before the study. In addition, confirmation of subject backgrounds, physical measurements, vital sign measurements, blood sampling for blood tests, urinalysis, gastrointestinal symptom rating scale (GSRS) score measurements, and a medical interview were performed. Sixteen subjects who met the eligibility criteria were enrolled and were randomly assigned to three groups, as described in Table 1. The subjects were assigned dynamically using the minimization method with BMI and age as the stratification factors.

### 2.3. Test Meals

The dietary interventions in this study used the following two diets and as well as the following two supplements. The subjects in the KD + placebo and KD + KF groups were provided a ketogenic diet based on a menu prepared by a registered dietitian. In addition, with each meal, the KD + KF group was provided a ketogenic formula (KF; Meiji Co., Ltd., Tokyo, Japan) and the KD + placebo group was provided a placebo formula. The ketogenic formula was a special infant formula with a high-fat, low-carbohydrate composition, which was formulated with MCT and had a ketogenic ratio of 3:1, which has long been used in Japan for ketogenic dietary therapy for infants with congenital metabolic disorders and refractory epilepsy. A placebo formula, in which the MCTs contained in the above KF were replaced with long-chain triglycerides. Three meals were provided per day on the test days, taking the estimated daily energy requirements of healthy adults, according to the 2015 Dietary Intake Standards (estimated daily energy requirements were 2300 kcal, and a daily energy intake of 1112 kcal was taken for the KF or placebo formula; a range of ±10% was allowed), into account. The energy balance of protein (P), fat (F), and carbohydrate (C) of the test meals in the KD + placebo and KD + KF groups was P:F:C = 22%:70%:8% (within ±5% was acceptable). The control group was provided a normal diet, taking the estimated daily energy requirements of healthy adults according to the 2015 Dietary Intake Standards into account. In addition, 200 mL of cow’s milk was provided to the control group instead of the ketogenic formula. The energy balance of protein (P), fat (F), and carbohydrate (C) of the test meal for the control group was P:F:C = 20%:23%:57% (within ±5% was acceptable). Eating rate was recorded, and each subject ingested the test meals in an isolated space so that they could not determine which meal was provided.

### 2.4. Study Design

**Figure 1:**
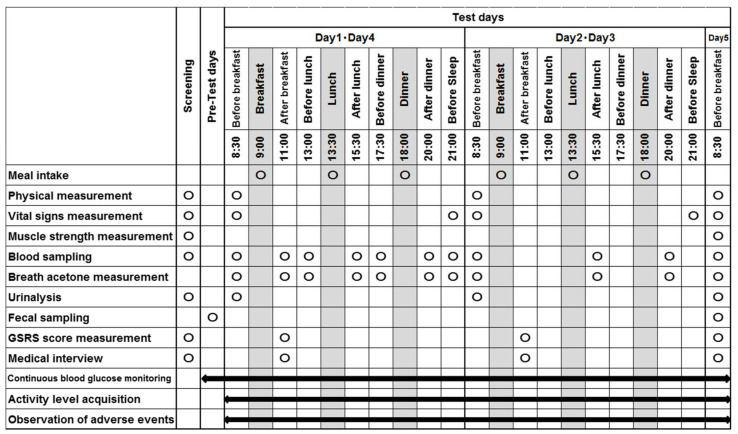
Exam schedule.

This study was conducted as a double blind, randomized, parallel-group comparative study. In this study, subjects were admitted to the Academic Clinical Research Center of Osaka University Hospital (Phase 1 Unit). The exam schedule is summarized in Figure 1. The subjects visited the hospital before the test day (Day 1), and muscle strength was measured and an intermittently scanned continuous glucose monitoring system was attached. In addition, fecal samples were collected on Day 1. Subjects visited the hospital on Day 1 without having eaten breakfast and were equipped with activity trackers to measure their activities. Subjects ingested the test meal for breakfast, after body composition, vital signs, and breath acetone were assessed, and blood sampling and urinalysis were performed. Two hours after breakfast, blood was collected, breath acetone and GSRS score were measured, and medical interviews were conducted. After that, the test meals were ingested for lunch and dinner. Blood sampling and breath acetone measurements were performed before and 2 h after the ingestion of the test meal. The measurements of vital signs and breath acetone, as well as blood sampling, were performed before bedtime.

On Day 2 and Day 3, assessments of body composition, vital signs and breath acetone, blood sampling, and urinalysis were performed before breakfast. Two hours after breakfast, GSRS scores were measured and medical interviews were conducted. Blood sampling and breath acetone measurements were performed 2 h after lunch and dinner. Vital signs were measured before bedtime. On Day 4, the same measurements as those of Day 1 were performed. Assessments of body composition, vital signs and breath acetone, blood sampling, and urinalysis were performed before breakfast. Subjects ingested breakfast, and 2 h later, blood sampling, breath acetone, and GSRS score measurements were taken and interviews were performed. After that, the test meals were ingested for lunch and dinner. Blood sampling and breath acetone measurements were performed before and 2 h after lunch, and before and 2 h after dinner. The measurement of vital signs and breath acetone, as well as blood sampling, were performed before bedtime.

On Day 5, body composition, vital signs, muscle strength, breath acetone, and GSRS scores were measured, blood and feces were collected, and urinalysis and medical interviews were performed before breakfast. Subjects spent time at the hospital and were prohibited from participating in hard exercise, smoking, and eating or drinking other than the food and drink provided by the hospital.

### 2.5. Experimental Measurements

#### 2.5.1. Physical Measurements

Body weight, body mass index (BMI), muscle mass, and body fat mass were measured using InBody (InBody Japan Inc., Tokyo, Japan). Height was measured only at screening. The measurement of vital signs included heart rate, blood pressure, and body temperature. Muscle strength measurements included grip strength, toe strength, and knee strength.

#### 2.5.2. Blood Test

On screening day and Day 5, blood samples were taken to evaluate hematology (white blood cells (WBC), red blood cells (RBC), hemoglobin (Hb), hematocrit (Ht), platelet count (PLT)), aspartate aminotransferase (AST), alanine aminotransferase (ALT), γ-glutamyl transferase (γGTP), alkaline phosphatase (ALP), blood urea nitrogen (BUN), creatinine (CRE), uric acid (UA), total-cholesterol, triglycerides (TG), HDL-cholesterol, LDL-cholesterol, albumin (ALB), transthyretin (TTR), blood glucose, glucagon (IRG), insulin (IRI), C-reactive protein (CRP), sodium (Na), potassium (K), chloride (CL), ketone bodies (acetoacetic acid, β-hydroxybutyrate), and medium-chain fatty acids (ALB was measured only at screening). During the test days (Day 1 to Day 4), plasma ketone body (acetoacetic acid and β-hydroxybutyrate) concentrations, plasma medium-chain fatty acid concentrations, and blood glucose were measured. For medium-chain fatty acid concentrations (octanoic acid (C8), decanoic acid (C10), and dodecanoic acid (C12)), plasma samples were methyl esterificated using a Fatty Acid Methylation Kit (Nacalai tesque, Kyoto, Japan) according to the manufacturer’s protocol. Methylated medium-chain fatty acids were quantified using GC-MS (SHIMADZU, Kyoto, Japan).

#### 2.5.3. Other Measurement

For expiratory acetone measurement, a breath acetone-measuring device (NTT docomo, Tokyo, Japan) was used. Urinalysis tests included urinary protein, glucose, and urobilinogen. Fecal samples were analyzed for gut microbiota and were measured by Cykinso Inc. (Tokyo, Japan). The QOL of gastrointestinal symptoms was measured using the gastrointestinal symptom rating scale (GSRS) score, which is a validated questionnaire regarding reflux, abdominal pain, indigestion, diarrhea, and constipation. Continuous blood glucose was monitored using a FreeStyle Libre Pro (Abbot Japan, Tokyo, Japan). For physical activity measurements, an ActiWatch (Philips Japan, Tokyo, Japan) was used.

#### 2.5.4. Gut Microbiota Analysis

Fecal sampling, DNA extraction, and sequencing were performed, as previously described [12]. Briefly, fecal samples were collected using brush-type collection kits containing guanidine thiocyanate solution (Techno Suruga Laboratory, Shizuoka, Japan), and were transported at ambient temperature and stored at 4 °C. DNA extraction from the fecal samples was performed using an automated DNA extraction system (GENEPREP STAR PI-480, Kurabo Industries Ltd., Osaka, Japan) according to the manufacturer’s protocols. The V1–V2 region of the 16S rRNA gene was amplified using a forward primer (16S_27Fmod: TCG TCG GCA GCG TCA GAT GTG TAT AAG AGA CAG AGR GTT TGA TYM TGG CTC AG) and a reverse primer (16S_338R: GTC TCG TGG GCT CGG AGA TGT GTA TAA GAG ACA GTG CTG CCT CCC GTA GGA GT) and KAPA HiFi HotStart ReadyMix (Roche, Basel, CHE) according to a previous study [13]. To sequence 16S amplicons using the Illumina MiSeq platform, dual index adapters were attached using the Nextera XT Index Kit. Each library was diluted to 5 ng/μL, and equal volumes of the libraries were mixed to 4 nM. The DNA concentration of the mixed libraries was quantified by qPCR with a KAPA SYBR FAST qPCR Master Mix (KK4601, KAPA Biosystems) using primer 1 (AAT GAT ACG GCG ACC ACC) and primer 2 (CAA GCA GAA GAC GGC ATA CGA). The library preparations were carried out according to the 16S library preparation protocol of Illumina (Illumina, San Diego, CA, USA). Libraries were sequenced using the MiSeq Reagent Kit v2 (500 Cycles) to produce 250-bp paired-end reads. Taxonomy assignment based on 16S rRNA gene sequences was carried out, as previously described [12].

### 2.6. Evaluation (Endpoints)

#### 2.6.1. Primary Outcome

Plasma ketone body (TKB) concentration represents the sum of acetoacetic acid (AcAc) concentration and β-hydroxybutyrate (BHB) concentration in plasma.

#### 2.6.2. Secondary Outcome

Secondary outcomes included plasma AcAc concentration, plasma BHB concentration, plasma medium-chain fatty acid concentration, and breath acetone concentration (BrAce).

#### 2.6.3. Safety Evaluation

Medical interviews, GSRS scores, weight, BMI, muscle mass, body fat mass, heart rate, blood pressure, body temperature, grip strength, toe strength, knee strength, hematology tests, AST, ALT, γGTP, ALP, BUN, CRE, UA, T-chol, TG, HDL-chol, LDL-chol, TTR, blood glucose, IRG, IRI, CRP, Na, K, CL, gut microbiota, urine protein, urine glucose, and urine urobilinogen were all used for safety assessments.

### 2.7. Statistical Analyses

The main analysis was as follows: two-way analysis of variance (ANOVA) was performed with the area under the curve (AUC) of plasma ketone body concentration per day on Day 1 and Day 4 as the objective variable, and groups (KD + KF and KD + placebo) and test days (Day 1, Day 4) were the explanatory variable. The secondary analysis was a two-way analysis of variance that was performed to evaluate the difference between groups and the difference between test days. This was done with AUC and maximum values (Cmax) of plasma TKB, AcAc, BHB, medium-chain fatty acid, and BrAce concentrations per day as the objective variables. When a significant difference was observed by ANOVA, Tukey–Kramer’s test was performed as a post hoc analysis. For the safety analysis, the frequency and grade of adverse events during the test days were aggregated.

## 3. Results

### 3.1. Participants

**Figure 2:**
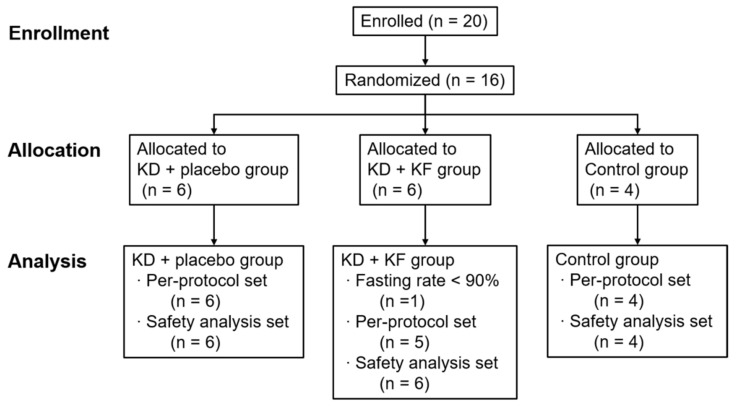
Flow diagram of research subjects. Abbreviations; KD, ketogenic diet; KF, ketogenic formula.

Twenty subjects were enrolled in this study. To calculate the sample size, the AUC of plasma ketone body concentration after a single administration of the ketogenic formula was estimated, based on a previous study [14]. In this study, the required number of subjects was 6 in the placebo and ketogenic groups, assuming that the difference between the two observed groups in the single-dose study was maintained for 12.5 h and that there was no accumulation of ketone bodies due to continuous administration. Of the 20 subjects who participated in the screening, 16 subjects were enrolled and randomly assigned to the 3 groups (Figure 2). In the baseline data, only albumin showed differences between groups, but there were no significant differences in the other background factors, such as height, weight, muscle mass, medical history, and blood data (Supplementary Tables S1 and S2). One subject in the KD + KF group had a dietary intake rate of 50% or less on two days, so he was excluded from analyses. In addition, the amount of activity during the test days showed a significant difference between the groups in terms of mean METs on Day 2; however, this was considered to be negligible because the mean difference was about 0.02 METs (Supplementary Table S3).

### 3.2. Primary Outcome

**Figure 3:**
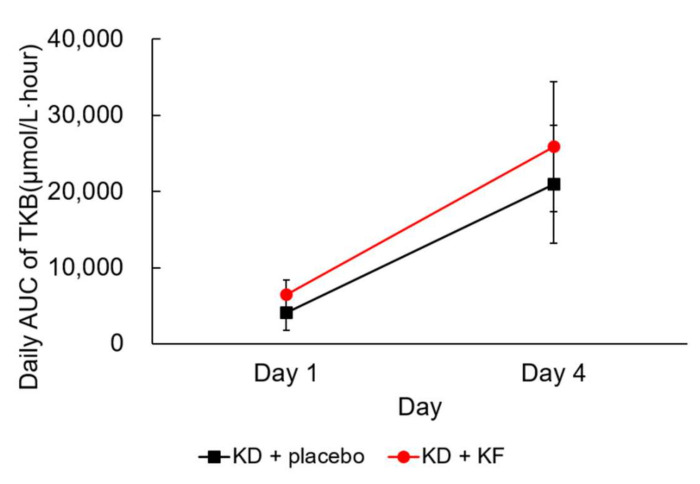
Changes in daily AUC for plasma TKB concentration. Values are presented as mean ± standard deviations. Abbreviations; AUC, area under the curve; TKB, total ketone body.

The daily AUC of plasma TKB concentration was significantly increased from Day 1 to Day 4 in both the KD + KF and KD + placebo groups (*p* = 0.004 and *p* < 0.001, respectively) (Figure 3, Table 2). However, there was no significant difference between groups, and no interaction was detected between the group and the test day (data not shown).

**Figure 4:**
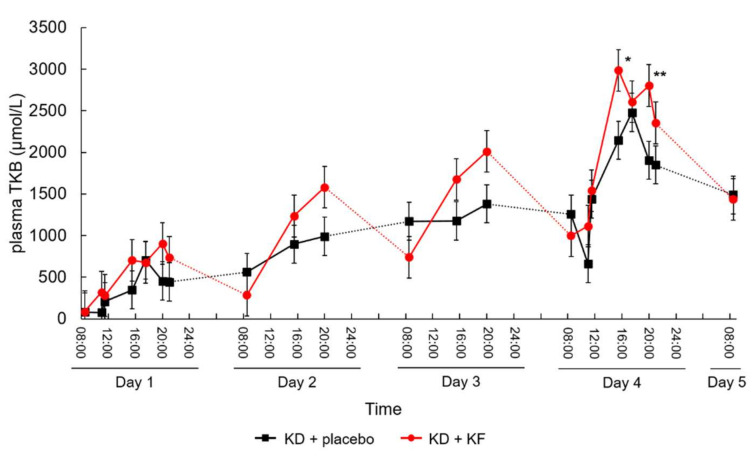
Changes in plasma TKB concentration over time. Subjects ingested the test meal at 9:00 a.m., 1:00 p.m. (13:30), and 6:00 p.m. (18:00) each day, and their blood was collected before and 2 h after ingesting the test meals. Values are presented as least square mean ± standard error. *: *p* < 0.05, **: *p* < 0.01, vs. KD + placebo group.

On the other hand, the diurnal variations in TKB concentration in the KD + KF group was larger than that in the KD + placebo group, and there was a statistically significant difference between the KD + KF and KD + placebo groups in terms of TKB concentrations after lunch and after dinner on Day 4 (Figure 4; *p* = 0.014 and *p* = 0.009, respectively).

### 3.3. Secondary Outcome

**Figure 5:**
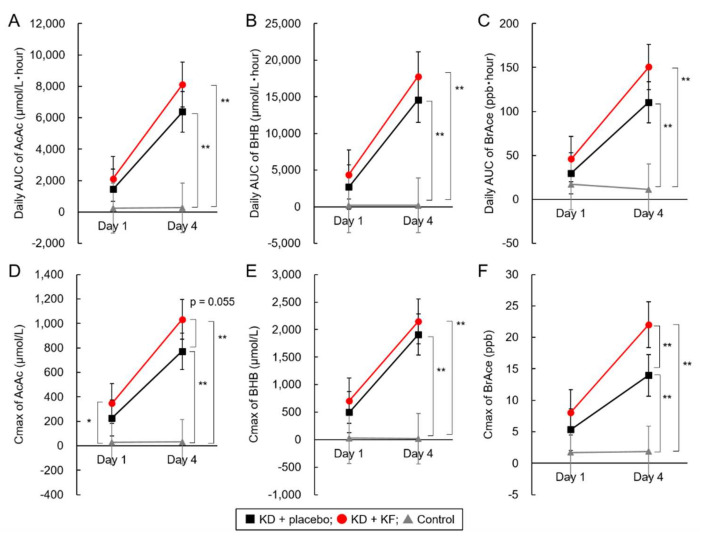
Analysis of the results of secondary endpoints. AUC of plasma AcAc (**A**), BHB (**B**), and breath acetone (**C**) concentrations, and the maximum concentration of plasma AcAc (**D**), BHB (**E**), and breath acetone (**F**). *: *p* < 0.05, **: *p* < 0.01, Tukey–Kramer’s test. All parameters in KD + placebo group and KD + KF group changed significantly between Day 1 and Day 4 (*p* < 0.05).

No significant changes due to dietary intervention were observed in the control group. In the KD + placebo and the KD + KF groups, the daily AUC and Cmax of plasma AcAc, plasma BHB, and BreAce concentrations showed a significant increase from Day 1 to Day 4 (Table 3, Figure 5A–F). In comparison with the control group, the KD + placebo and KD + KF groups were significantly different in terms of daily AUC and Cmax of plasma AcAc, plasma BHB, and BreAce concentrations at Day 4. However, there were no significant differences between the KD + placebo and the KD + KF groups for all secondary endpoints, except in the Cmax of BrAce (Figure 5; *p* = 0.007). The diurnal variation of plasma BHB concentration and BrAce showed similar tendencies in the KD + KF group and the KD + placebo group (Supplementary Figure S1).

**Figure 6:**
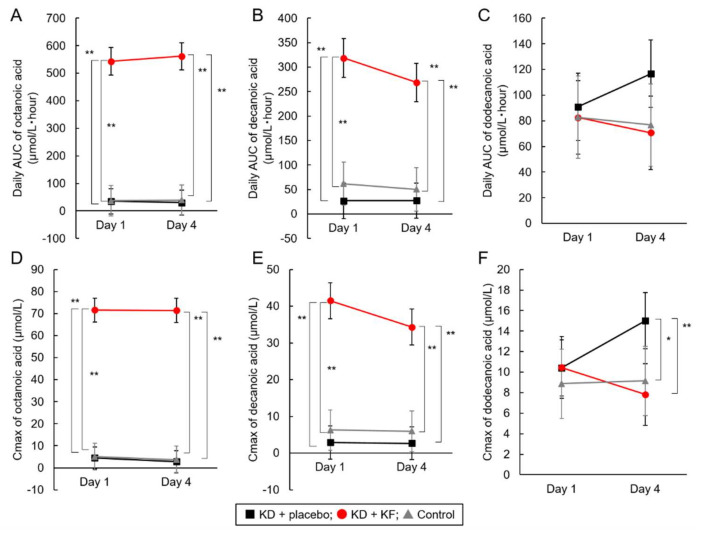
Analysis of the results of secondary endpoints. The daily AUC of plasma octanoic (**A**), decanoic (**B**), dodecanoic (**C**) acid concentration; Cmax of plasma octanoic (**D**), decanoic (**E**), dodecanoic (**F**) acid concentration. *: *p* < 0.05, **: *p* < 0.01, Tukey–Kramer’s test.

The daily AUC and Cmax of plasma octanoic and decanoic acid levels were already high at Day 1 and did not increase significantly from Day 1 to Day 4 in the KD + KF group (Table 3 and Figure 6A–F). In addition, significant differences between the KD + KF group and the other two groups were observed in the daily AUC and Cmax of octanoic and decanoic acid in plasma (*p* < 0.01). The Cmax of dodecanoic acid in the KD + placebo group was significantly higher than that in the other two groups at Day 4. There were no significant differences in the daily AUC of plasma dodecanoic acid concentrations between groups, and no interactions were detected between the group and the test day (Supplementary Table S6).

The plasma octanoic and decanoic acid concentrations in the KD + KF group and the plasma dodecanoic acid concentration in the KD + placebo group remained significantly high (Figure 6). The main components of medium-chain triglycerides that were abundant in KF are octanoic and decanoic acids. On the other hand, the placebo formula did not contain MCT, but the triglycerides blended in the placebo formula contained a tiny amount of dodecanoic acid. These data were due to the dietary administration.

### 3.4. Safety Analysis

No adverse events were observed in the control group (Table 4). In the KD + KF group, hyperuricemia, hyperlipidemia, and upper abdominal pain were observed in three, one, and one subjects, respectively (Table 4). In the KD + placebo group, hyperuricemia was observed in three subjects. While a causal association with the test meal could not be ruled out, all were mild cases and recovered without treatment (Table 4). There was no significant difference in the number of adverse events between the KD + KF group and the KD + placebo group. Therefore, there were no safety problems, at least for short-term KF intake.

### 3.5. Physical Measurement and Vital Signs

In the KD + placebo group, body weight, BMI, body fat mass, right leg muscle mass, and left leg muscle mass decreased significantly from Day 1 to Day 5 (Supplementary Table S4). In the KD + KF group, body weight, BMI, body fat mass, muscle mass, left arm muscle mass, trunk muscle mass, and right leg muscle mass were significantly reduced. In the control group, body weight and BMI were significantly reduced. However, no differences were observed between the physical parameters of the groups. Therefore, the loss of muscle mass and body weight were likely due to confinement for a certain period of time and the decrease in food intake. Although a significant decrease in muscle mass was observed in the KD + placebo and KD + KF groups, the average decrease was about 3%, suggesting that safety concerns were minimal. However, periodic monitoring of body composition, including muscle mass is required for long-term intake of KF. On the other hand, the average body fat mass of the KD + placebo group and the KD + KF group decreased by about 5% or more, so the ketogenic diet has the potential to lead to weight loss (Supplementary Table S4).

There were no significant differences in the vital signs among the three groups, and vital signs varied within the normal range during the test days for all groups (Supplementary Table S5). In other words, these changes were likely due to captured physiological diurnal variations, and it was considered that there was no particular safety problem.

### 3.6. Muscle Strength

From the screening day to Day 5, knee strength was significantly increased in the KD + placebo group, and right toe strength was significantly increased in the KD + KF group. However, no differences were observed between the groups in terms of muscle strength. Therefore, there was no particular safety problem regarding muscle strength. It seems contradictory that muscle strength increased even though muscle mass decreased; however, subjects were likely to apply greater strength during the measurement on Day 5 because of the learning effect on the measurement of the muscle strength (Supplementary Table S6).

### 3.7. Blood Parameters

In the KD + placebo group, PLT (*p* = 0.029), ALP (*p* = 0.014), TTR (*p* = 0.002), blood glucose (*p* = 0.005), and IRI (*p* = 0.008) decreased significantly, and BUN (*p* = 0.022), UA (*p* < 0.001), and IRG (*p* = 0.026) showed significant increases from baseline to Day 5 (Table 5). In the KD + KF group, TTR (*p* = 0.007), blood glucose (*p* = 0.006), and IRI (*p* = 0.011) were significantly decreased, and BUN (*p* < 0.001) and UA (*p* = 0.002) were significantly increased. In the control group, only IRI (*p* = 0.046) was significantly increased. On Day 5, UA (*p* < 0.001), TG (*p* = 0.004), TTR (*p* = 0.007), blood glucose (*p* < 0.001), and IRI (*p* < 0.001) showed significant differences between the groups. From these results, it was determined that a ketogenic diet increases UA and TG, decreases TTR, which is an indicator for ALB synthesis, and decreases blood glucose and IRI levels.

### 3.8. Urinalysis

No remarkable changes were observed for urine protein, urobilinogen, and other urinalyses during the test days. Therefore, no safety problems due to the ingestion of the test meals were detected regarding urinalysis.

### 3.9. Metabolic Parameters

There was a significant difference between the groups in the mean blood glucose levels determined using continuous blood glucose monitoring (*p* = 0.009). However, the time ratio below the reference value (70 mg/dL) showed no differences between groups (*p* = 0.799). The average blood glucose levels of the KD + placebo and KD + KF groups were lower than those of the control group because they ingested ketogenic diets, but this did not lead to an increase in hypoglycemic time. Moreover, the proportion of hypoglycemia in the KD + KF group was the lowest among the groups. Therefore, a ketogenic diet using KF was shown to decrease, but not to significantly fluctuate, blood glucose levels (Table 6).

### 3.10. Gut Microbiota

**Figure 7:**
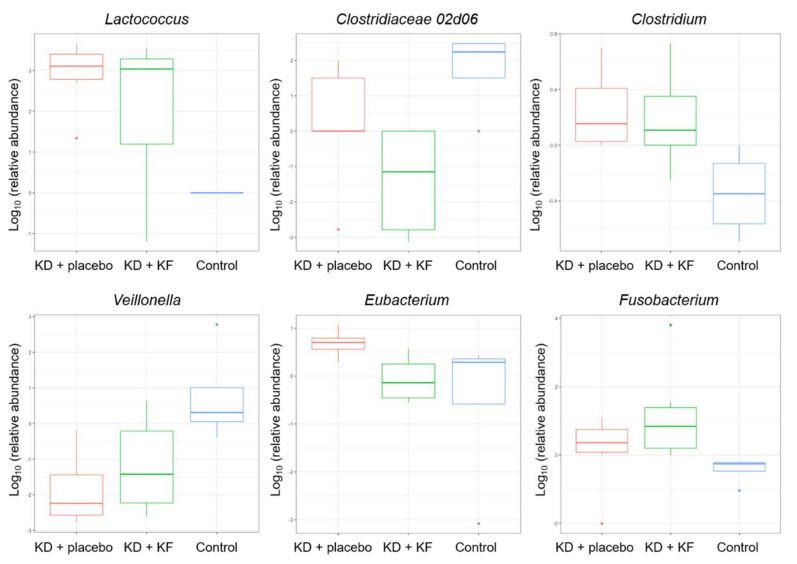
Comparison of gut microbiota composition between groups. Red bar, KD + placebo group; green bar, KD + KF group; blue bar, control group.

As a result of comparing the amount of change in the gut microbiota, six genera showed significant differences between groups (*Lactococcus*, *Clostridiaceae 02d06*, *Clostridium*, *Veillonella*, *Eubacterium*, *Fusobacterium*) (*p* < 0.05, Figure 7).

The similarity in the composition of gut microbiota was calculated as the Aitchison distance. The day of fecal sampling did not significantly affect the Aitchison distance in any groups (KD + placebo: R^2^ = 0.069, *p* = 0.788, KD + KF: R^2^ = 0.059, *p* = 0.908, control: R^2^ = 0.072, *p* = 1.000). These results indicated that the dissimilarity in gut microbiota was influenced by the individual differences at the same time points, which were greater than the differences between the time points within the same individual. The effect of the ketogenic diet on microbiota was shown to be not be large enough to change bacterial composition across the differences among the individuals or groups (Supplementary Figure S2). Diversity (Shannon index) tended to increase in the KD + placebo and KD + KF groups (Supplementary Figure S3). In addition, the *Firmicutes* to *Bacteroidetes* (FB) ratio, which has been suggested to be associated with obesity [15,16], tended to show a decrease in the phylum *Firmicutes*, though only in the KD + KF group (Supplementary Figure S4).

## 4. Discussion

The ketogenic diet requires a rigorous limitation of carbohydrates while allowing a liberal ingestion of fats (including saturated fats) and has generated intense medical debate. Clinical studies on ketogenic diets show promise for a variety of clinical indications; however, they often do not utilize consistent and standardized diets or compliance measures in order to enable the reproducibility and generalizability of study outcomes [17,18]. The current consensus is that the ketogenic diet is a potentially safe and powerful therapeutic intervention; however, it should be assessed on an individual basis, keeping in mind safety recommendations [18,19]. In overweight/obese individuals, it is a promising option to achieve a significant weight loss in a short period of time [20]. There is no clear evidence for advantages of ketogenic diets regarding cardiometabolic risk markers, such as low-density lipoprotein cholesterol levels [19,21]. There is currently conflicting evidence of the utility of a ketogenic diet in cancer therapy. It might be argued that the ketogenic diet, in combination with standard therapies, has the potential to enhance the antitumor effects of classic chemo- and radiotherapy, with an overall good safety and tolerability, as well as an increase in quality of life [22]. However, a recent systematic review and meta-analysis of randomized controlled trials revealed inadequate evidence to support the beneficial effects of a ketogenic diet in antitumor therapy [23]. Moreover, hyperuricemia, hyperlipidemia, and upper abdominal pain have been observed in previous studies with cancer patients [11,22,23]. Therefore, this was likely an adverse event that occurs when a ketogenic diet is administered. On the other hand, a low carbohydrate ketogenic diet could be beneficial to increase the quality of life, physical performance, body composition, and metabolic health of breast cancer patients [24], and it is well tolerated by glioblastoma patients [25]. To further elucidate the mechanisms of the ketogenic diet as a therapy, and to evaluate its application in clinical practice, more studies, as well as uniformly controlled clinical trials, are strongly needed.

In our double-blinded, randomized, parallel-group comparative study, the usefulness of standardized multiple doses of KF in ketone-body induction was examined. Compared with the KD + placebo group, the diurnal variation of total ketone bodies was significantly increased in the KD + KF group after lunch and after dinner on the 4th day. Although the sample size was limited, these results show that it might be necessary to focus on diurnal variation rather than daily AUC in order to evaluate the effects of KF on plasma TKB concentrations. Similar results were obtained for plasma AcAc and BHB concentrations. AcAc and BHB are robust readouts of KF administration, and supply of these exogenous ketone bodies and ketone sources can even be considered a complement to, or an alternative to, the ketogenic diet itself [26]. It has been previously shown that ketonuria in subjects with stable ketosis is highest and can be most reliably detected in urine at certain times of the day, such as post-dinner [27], which is consistent with our findings. This can provide recommendations regarding the precise time of the day for measuring ketone bodies. We suggest that KF may be useful for inducing the peak of ketone bodies. Since it has been reported that induction of ketone bodies affects clock genes [28], it is possible that the difference between the peak value and the trough value of ketone bodies may affect the long-term prognosis of cancer patients. In the KD + KF group, the maximum value of BrAce was significantly higher than that in the KD + placebo group. This may indicate that the induction of ketone bodies by medium-chain fatty acids (octanoic and decanoic) is different from the induction of ketone bodies by long-chain fatty acids, or even by another medium-chain fatty acid (dodecanoic).

In addition, TTR decreased and blood glucose and IRI levels decreased in both the KD + KF and the KD + placebo groups; however, IRG did not show a significant increase in the KD + KF group, and, even in continuous blood glucose monitoring, the proportion of hypoglycemia in the KD + KF group was the lowest among the three groups. Hence, the effect of KF on glucose metabolism may be different from that of the placebo. These questions will need to be examined through further metabolomic analyses. Considering energy metabolism, a study on the effects of 20 days of ketogenic diet on metabolic and respiratory parameters in healthy subjects uncovered that a ketogenic diet, overall, decreased rest energy expenditure, decreased carbon dioxide body stores, and there was a higher percentage of fat loss versus a standard Mediterranean diet [29]. We observed that the KD + KF group displayed a paradoxical increase in muscle strength, although muscle mass was decreased, which might be dependent on the type of muscle analyzed [30].

In the KD + KF group, three subjects experienced mild hyperuricemia, one subject showed hyperlipidemia, and one subject presented with upper abdominal pain. However, all cases recovered without treatment according to subsequent follow up. These are possible adverse events when a ketogenic diet is ingested, which we previously observed in past clinical trials [11,14]. In addition, there was no major difference in the number of adverse events for which a causal association cannot be ruled out in the KD + KF and KD + placebo groups. These results suggest there are no safety issues related to KF intake.

Ketogenic diets can have a dramatic impact on human microbiota in a very distinct manner compared to a high-fat diet. In vitro and in vivo experiments have shown that ketone bodies selectively inhibited bifidobacterial growth [31]. Moreover, ketogenic diet-associated gut microbiota reduced the levels of intestinal pro-inflammatory Th17 cells [31]. Dysbiosis of the gut microbiota could be involved in the pathogenesis of epilepsy, which can be relieved by a ketogenic diet [32]. Other human and animal studies have shown controversial effects in reshaping bacterial architecture and gut biological functions, such as a lowered diversity and an increased amount of pro-inflammatory bacteria [33]. Although further analysis is needed to investigate a correlation between certain bacterial species and blood parameters, such as that done in a previous study [34], KF did not significantly influence gut microbiota composition, suggesting a neutral and safe effect.

The main limitation of our study is the relatively small sample size and the short term of the study period, which might not have clearly revealed the effects of KF on several metabolic pathways and on gut microbiota composition. Moreover, another limitation is that the subjects in this study were not elderly people. In clinical practice, it is very important to reveal the effects of a ketogenic diet on geriatric syndromes. A larger amount of baseline data from elderly subjects is needed to accumulate information about the effects of the ketogenic diet and the formulations of ketogenic diet regimens. In our pilot human study, we reported that multiple doses of a ketogenic diet incorporating KF displays metabolic and biochemical safety and tolerability. This information might be useful to standardize and to safely practice ketogenic diets in clinical applications, such as for cancer therapy.

## Figures and Tables

**Table 1:** Group compositions.

Group	Test Meal
KD + placebo (P group; N = 6)	Ketogenic diet + placebo formula 50 g (200 mL) Taken 3 times a day
KD + KF (K group; N = 6)	Ketogenic diet + KF 50 g (200 mL) Taken 3 times a day
Control (C group; N = 4)	Normal diet + cow’s milk (200 mL) Taken 3 times a day

**Table 2:** Comparison of daily AUC of plasma TKB concentration between Day 1 and Day 4.

AUC of Plasma Ketone Body (μmol/L∙h)	Day 1	Day 4	Comparison between Day 1 and Day 4 (*p* Value)
KD + placebo	4130 ± 2393	20,968 ± 7708	<0.001
KD + KF	6511 ± 1893	25,891 ± 8493	0.004

Values are presented as mean ± standard deviations.

**Table 3:** Comparison of secondary endpoints, before and after intervention.

Metabolites		Group	Day 1	Day 4	Comparison between Day 1 and Day 4 (*p* Value)
AUC	AcAc	KD + placebo	1442 ± 727	6381 ± 2100 ^a^	<0001
		KD + KF	2109 ± 546	8116 ± 2774 ^a^	0.005
		Control	244 ± 54	267 ± 40 ^b^	0.238
	BHB	KD + placebo	2688 ± 1687	14,587 ± 5673 ^a^	<0.001
		KD + KF	4402 ± 1357	17,775 ± 5737 ^a^	0.004
		Control	200 ± 49	185 ± 31 ^b^	0.361
	Octanoic Acid	KD + placebo	34.8 ± 11.1 ^a^	30.1 ± 38.7 ^a^	0.698
		KD + KF	543.5 ± 104.8 ^b^	561.9 ± 52.4 ^b^	0.643
		Control	36.9 ± 2.7 ^a^	39.6 ± 43.4 ^a^	0.903
	Decanoic Acid	KD + placebo	26.9 ± 25.6 ^a^	27.5 ± 36.1 ^a^	0.974
		KD + KF	318.5 ± 37.8 ^b^	268.6 ± 73.5 ^b^	0.099
		Control	62.0 ± 30.7 ^a^	50.3 ± 36.4 ^a^	0.556
	Dodecanoic Acid	KD + placebo	90.9 ± 29.5	116.7 ± 38.5	0.182
		KD + KF	82.6 ± 21.1	70.7 ± 37.3	0.449
		Control	82.8 ± 27.9	76.8 ± 24.4	0.707
	BrAce	KD + placebo	29.8 ± 17.2	110.4 ± 32.0 ^a^	<0.001
		KD + KF	46.1 ± 6.4	150.6 ± 52.0 ^a^	0.012
		Control	17.2 ± 18.7	11.3 ± 4.3 ^b^	0.596
Cmax	AcAc	KD + placebo	226 ± 113 ^a^	772 ± 213 ^a^	<0.001
		KD + KF	347 ± 116 ^b^	1032 ± 314 ^b^	0.002
		Control	27 ± 7 ^a^	31 ± 5 ^a^	0.231
	BHB	KD + placebo	497 ± 335	1911 ± 687 ^a^	0.002
		KD + KF	707 ± 190	2149 ± 648 ^a^	0.003
		Control	28 ± 9	20 ± 2 ^b^	0.103
	Octanoic Acid	KD + placebo	4.5 ± 1.9 ^a^	2.8 ± 3.0 ^a^	0.309
		KD + KF	71.6 ± 12.0 ^b^	71.5 ± 6.2 ^b^	0.973
		Control	5.1 ± 2.5 ^a^	3.7 ± 3.4 ^a^	0.529
	Decanoic Acid	KD + placebo	2.9 ± 2.6 ^a^	2.7 ± 2.9 ^a^	0.880
		KD + KF	41.5 ± 8.4 ^b^	34.3 ± 8.3 ^b^	0.156
		Control	6.3 ± 2.6 ^a^	6.0 ± 3.0 ^a^	0.845
	Dodecanoic Acid	KD + placebo	10.4 ± 3.3	15.0 ± 4.8 ^a^	0.077
		KD + KF	10.4 ± 2.3	7.8 ± 2.9 ^b^	0.168
		Control	8.9 ± 2.6	9.2 ± 2.1 ^b^	0.882
	BrAce	KD + placebo	5.4 ± 3.1	14.0 ± 4.8 ^a^	0.002
		KD + KF	8.1 ± 2.0	22.0 ± 6.8 ^b^	0.011
		Control	1.7 ± 1.4	1.9 ± 0.8 ^c^	0.858

Values are presented as mean ± standard deviations, and values with different superscripts are significantly different (*p* < 0.05).

**Table 4:** Safety assessment.

	KD + Placebo (N = 6)	KD + KF (N = 6)	Control (N = 4)
Metabolic and nutritional disorders			
Hyperuricemia	3 (50.0%)	3 (50.0%)	0 (0.0%)
Hyperlipidemia	0 (0.0%)	1 (16.7%)	0 (0.0%)
Gastrointestinal disorders			
Upper abdominal pain	0 (0.0%)	1 (16.7%)	0 (0.0%)

**Table 5:** Blood test analyses.

	KD + Placebo (N = 6)	KD + KF (N = 6)	Control (N = 4)	Comparison between Groups (*p* Value)
White blood cells, ×10^3^/μL				
Baseline	6.380 ± 1.942	5.953 ± 1.174	4.855 ± 0.755	0.293
Day 5	5.295 ± 1.309	5.643 ± 1.024	5.212 ± 0.709	0.791
Red blood cells, ×10^6^/μL				
Baseline	5.293 ± 0.291	5.177 ± 0.241	5.420 ± 0.415	0.489
Day 5	5.423 ± 0.091	5.260 ± 0.219	5.282 ± 0.512	0.589
Hemoglobin, g/dL				
Baseline	15.63 ± 0.96	15.43 ± 0.70	15.72 ± 0.75	0.844
Day 5	16.02 ± 0.45	15.80 ± 0.99	15.55 ± 0.72	0.642
Hematocrit, %				
Baseline	47.00 ± 1.87	46.58 ± 1.74	48.08 ± 2.28	0.498
Day 5	47.35 ± 1.25	46.83 ± 2.59	46.48 ± 2.94	0.832
Platelet count, ×10^3^/μL				
Baseline	278.7 ± 59.1	261.8 ± 34.4	252.8 ± 40.2	0.673
Day 5	232.0 ± 56.8 *	265.2 ± 41.0	247.5 ± 48.9	0.525
AST, U/L				
Baseline	17.7 ± 3.9	18.2 ± 1.6	20.2 ± 2.6	0.394
Day 5	18.5 ± 4.0	18.2 ± 3.2	20.0 ± 3.8	0.732
ALT, U/L				
Baseline	16.5 ± 6.1	22.3 ± 11.9	19.8 ± 8.1	0.556
Day 5	15.7 ± 5.6	21.2 ± 13.8	21.5 ± 14.6	0.651
γGTP, U/L				
Baseline	21.3 ± 6.3	26.7 ± 16.3	19.5 ± 7.2	0.582
Day 5	18.3 ± 4.0	23.2 ± 15.5	18.2 ± 8.4	0.688
ALP, U/L				
Baseline	209.8 ± 37.3	241.3 ± 57.0	254.0 ± 57.8	0.376
Day 5	190.8 ± 35.3 *	236.3 ± 53.2	243.0 ± 41.2	0.145
BUN, mg/dL				
Baseline	12.0 ± 2.5	11.7 ± 2.1	12.2 ± 3.3	0.938
Day 5	16.7 ± 2.4 *	15.2 ± 2.6 **	13.2 ± 2.6	0.156
Creatinine, mg/dL				
Baseline	0.845 ± 0.099	0.770 ± 0.053	0.838 ± 0.048	0.200
Day 5	0.883 ± 0.074	0.812 ± 0.075	0.883 ± 0.075	0.220
Uric acid, mg/dL				
Baseline	5.57 ± 1.00	6.12 ± 0.93	5.70 ± 0.70	0.571
Day 5	8.50 ± 1.14 **	9.38 ± 1.30 **	5.83 ± 0.39	< 0.001
Total cholesterol, mg/dL				
Baseline	167.5 ± 23.1	177.0 ± 20.0	183.5 ± 24.0	0.533
Day 5	180.5 ± 10.3	183.0 ± 45.6	174.8 ± 26.7	0.921
TG, mg/dL				
Baseline	66.0 ± 16.9	99.3 ± 56.6	66.5 ± 15.7	0.265
Day 5	50.3 ± 10.3	55.8 ± 14.0	91.2 ± 24.8	0.004
HDL cholesterol, mg/dL				
Baseline	52.8 ± 8.2	52.8 ± 12.4	63.5 ± 11.4	0.264
Day 5	50.8 ± 11.2	52.8 ± 10.0	59.0 ± 9.4	0.482
LDL cholesterol, mg/dL				
Baseline	108.8 ± 25.4	109.2 ± 13.1	110.2 ± 20.5	0.994
Day 5	121.5 ± 9.9	114.7 ± 44.5	103.8 ± 18.5	0.659
Albumin, g/dL				
Baseline	4.75 ± 0.12	5.03 ± 0.18	4.80 ± 0.08	0.009
Day 5	-	-	-	
Transthyretin, mg/dL				
Baseline	31.40 ± 4.26	34.77 ± 4.65	35.02 ± 3.99	0.334
Day 5	23.95 ± 1.94 **	28.17 ± 5.89 **	35.80 ± 5.73	0.007
Glucose, mg/dL				
Baseline	84.7 ± 3.5	88.0 ± 4.9	87.8 ± 1.3	0.287
Day 5	74.0 ± 3.4 **	77.0 ± 5.0 **	89.5 ± 2.4	<0.001
Glucagon, pg/mL				
Baseline	128.5 ± 16.5	129.3 ± 24.5	142.8 ± 6.1	0.454
Day 5	167.8 ± 24.8 *	153.7 ± 41.8	138.8 ± 13.7	0.369
Insulin, μIU/mL				
Baseline	4.95 ± 1.63	5.07 ± 2.34	4.50 ± 1.05	0.887
Day 5	2.28 ± 1.05 **	2.45 ± 0.79 *	5.38 ± 0.62 *	< 0.001
CRP, mg/dL				
Baseline	0.030 ± 0.017	0.023 ± 0.008	0.020 ± 0.000	0.394
Day 5	0.045 ± 0.061	0.050 ± 0.060	0.020 ± 0.000	0.669
Na, mEq/L				
Baseline	4.27 ± 0.22	4.10 ± 0.14	4.25 ± 0.26	0.345
Day 5	4.37 ± 0.19	4.30 ± 0.24	4.22 ± 0.35	0.694
Cl, mEq/L				
Baseline	102.0 ± 1.4	102.0 ± 1.7	102.5 ± 0.6	0.825
Day 5	100.2 ± 2.5	100.8 ± 1.7	102.2 ± 2.2	0.352

Values are presented as mean ± standard deviations. *: *p* < 0.05, **: *p* < 0.01, vs. baseline, paired *t*-test.

**Table 6:** Analysis of continuous blood glucose monitoring.

	KD + Placebo (N = 6)	KD + KF (N = 6)	Control (N = 4)	Comparison between Groups (*p* Value)
Average of blood glucose (mg/dL)	86.18 ± 2.52	85.40 ± 4.12	95.55 ± 7.10	0.009
Percentage of hour when blood glucose was less than normal range (%)	2.70 ± 2.20	1.74 ± 2.58	3.21 ± 5.90	0.799

Values are presented as mean ± standard deviations.

## Data Availability

Data sharing not applicable.

## Supplementary Materials

The following are available online at https://www.mdpi.com/article/10.3390/nu14061199/s1, Supplemental Figure S1. Changes in the secondary endpoints; Supplemental Figure S2. The similarity of the composition of gut microbiota; Supplemental Figure S3. Alteration of the Shannon index of gut microbiota; Supplemental Figure S4. F/B ratio; Supplemental Table S1. Subjects’ characteristics of anthropometric measurements at screening; Supplemental Table S2. Subjects’ characteristics of safety measurements at screening; Supplemental Table S3. Analysis of activity measurements; Supplemental Table S4. Analysis of body measurements; Supplemental Table S5. Analysis of vital signs; Supplemental Table S6. Analysis of muscle strength.
