# Supplementary material for: Ketogenic Effects of Multiple Doses of a Medium Chain Triglycerides Enriched Ketogenic Formula in Healthy Men under the Ketogenic Diet: A Randomized, Double-Blinded, Placebo-Controlled Study
Source: Nutrients. 2022 Mar 12;14(6):1199. doi: 10.3390/nu14061199 (PMC8955388; doi:10.3390/nu14061199)

Supplemental Figure 1. Changes in the secondary endpoints

Values are presented as least square mean ± standard error. BB: before breakfast, AB: after breakfast, BL: before lunch, AL: after lunch, BD: before dinner, AD: after dinner, BS: before sleep. ■: KD + placebo, ●: KD + KF group. *: $p < 0.05$, **: $p < 0.01$, vs. KD + placebo group.

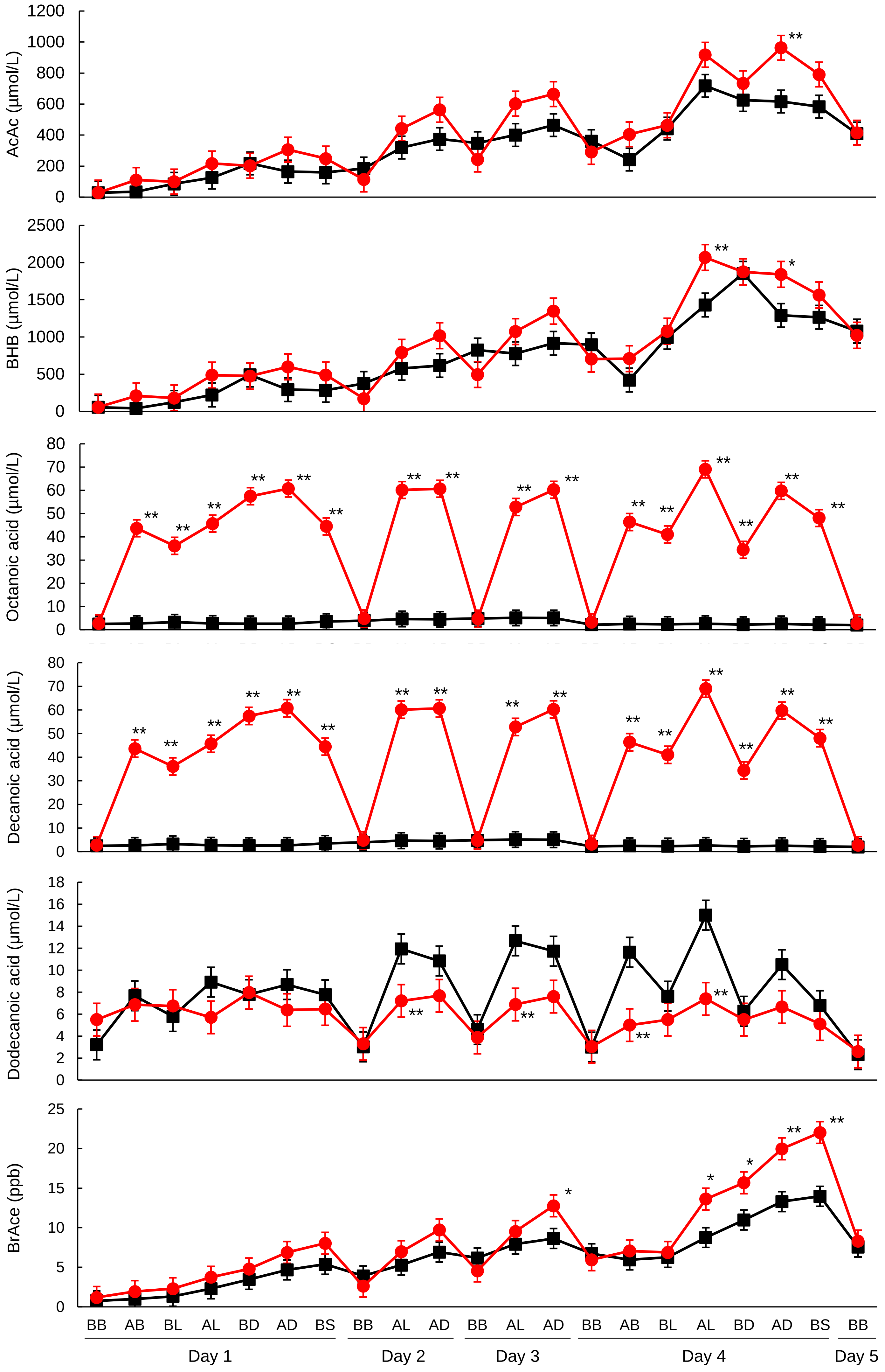

Supplement: Supplementary file 1 [file nutrients-14-01199-s001.zip › Supplemental_Figure_1.pdf]
