# Supplementary material for: Ketogenic Effects of Multiple Doses of a Medium Chain Triglycerides Enriched Ketogenic Formula in Healthy Men under the Ketogenic Diet: A Randomized, Double-Blinded, Placebo-Controlled Study
Source: Nutrients. 2022 Mar 12;14(6):1199. doi: 10.3390/nu14061199 (PMC8955388; doi:10.3390/nu14061199)

Supplemental Figure 2. The similarity of the composition of gut microbiota

The similarity of the composition of gut microbiota was calculated as the Aitchison distance, and visualized by the principal coordinates analysis method according to Aitchison distance. (a) KD + placebo, (b) KD + KF, and (c) control group. The similarity of the change in the gut microbiota between Day 1 and Day 5 was also calculated as the Aitchison distance and visualized (d).

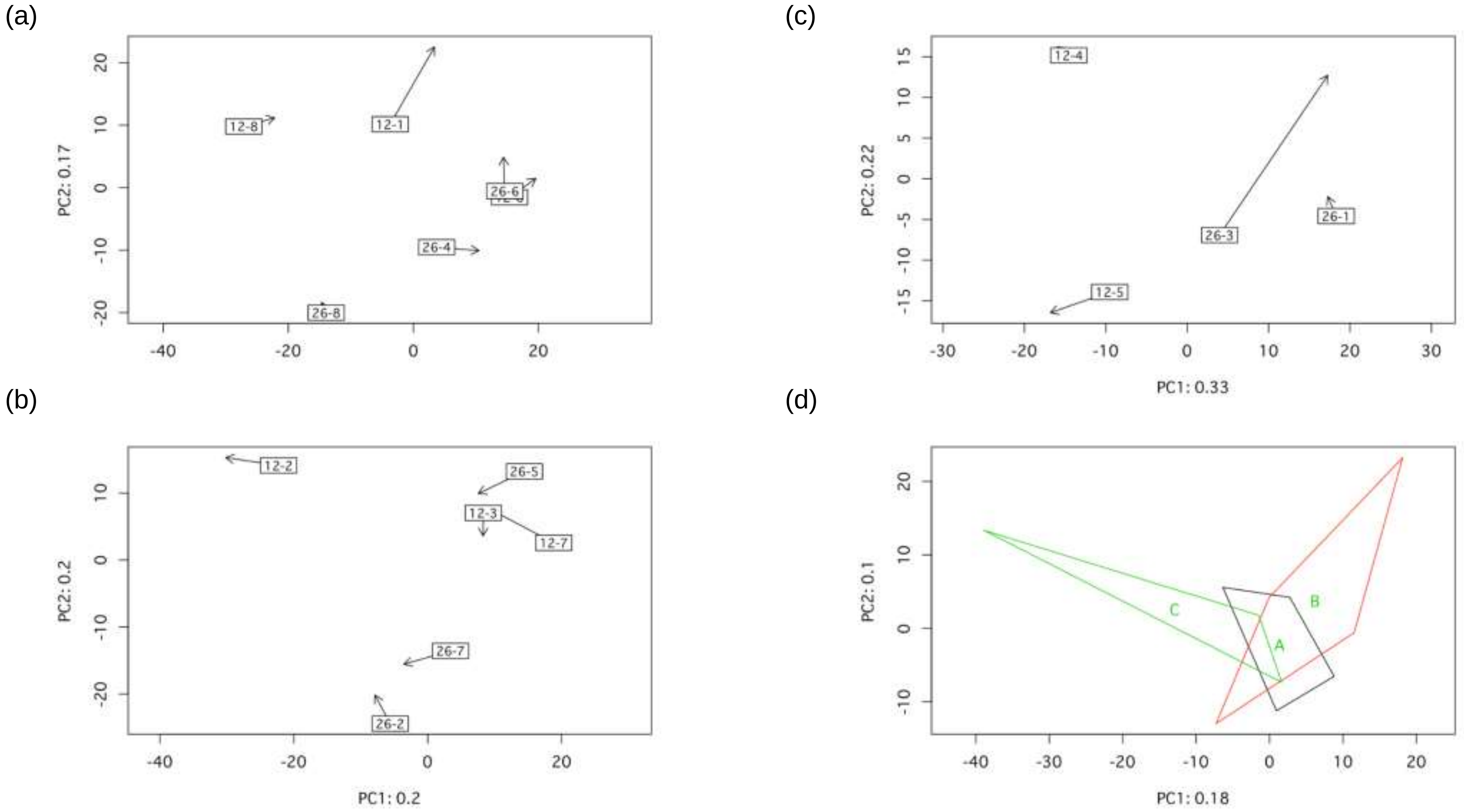

Supplement: Supplementary file 1 [file nutrients-14-01199-s001.zip › Supplemental_Figure_2.pdf]
