# Supplementary material for: Ketogenic Effects of Multiple Doses of a Medium Chain Triglycerides Enriched Ketogenic Formula in Healthy Men under the Ketogenic Diet: A Randomized, Double-Blinded, Placebo-Controlled Study
Source: Nutrients. 2022 Mar 12;14(6):1199. doi: 10.3390/nu14061199 (PMC8955388; doi:10.3390/nu14061199)

Supplemental Figure 3. Alteration of the Shannon index of gut microbiota

Microbiota diversity was assessed by the Shannon index based on 97% nucleotide sequence identity.
microbiota diversity indices (Shannon index) tended to increase in the KD + placebo and the KD + KF group.
no interaction was detected between the group and the test day (p = 0.293).

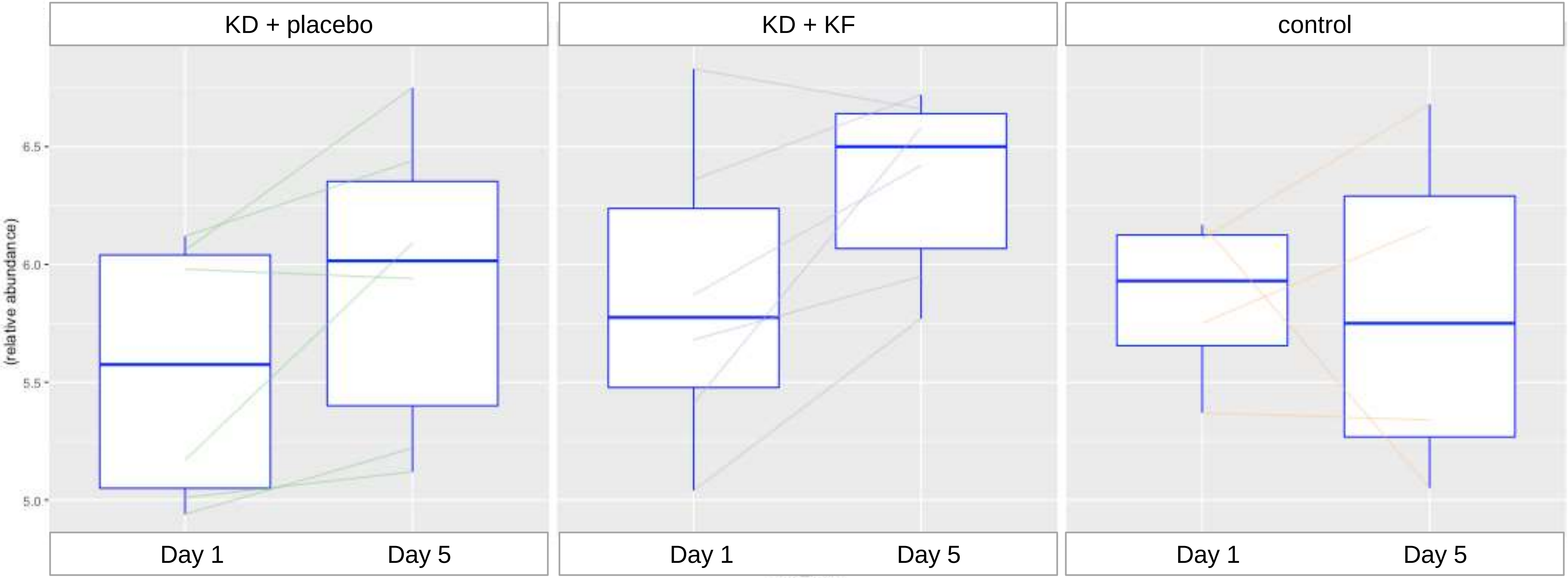

Supplement: Supplementary file 1 [file nutrients-14-01199-s001.zip › Supplemental_Figure_3.pdf]
