# Supplementary material for: Ketogenic Effects of Multiple Doses of a Medium Chain Triglycerides Enriched Ketogenic Formula in Healthy Men under the Ketogenic Diet: A Randomized, Double-Blinded, Placebo-Controlled Study
Source: Nutrients. 2022 Mar 12;14(6):1199. doi: 10.3390/nu14061199 (PMC8955388; doi:10.3390/nu14061199)

Supplemental Figure 4. FB ratio

The FB ratio is the ratio of the phylum *Firmicutes* to the phylum *Bacteroidetes*.
The phylums *Firmicutes* tend to decrease only in the KD +KF group.
the result showed a significant difference in interaction effects between the group and the test day (p = 0.017)

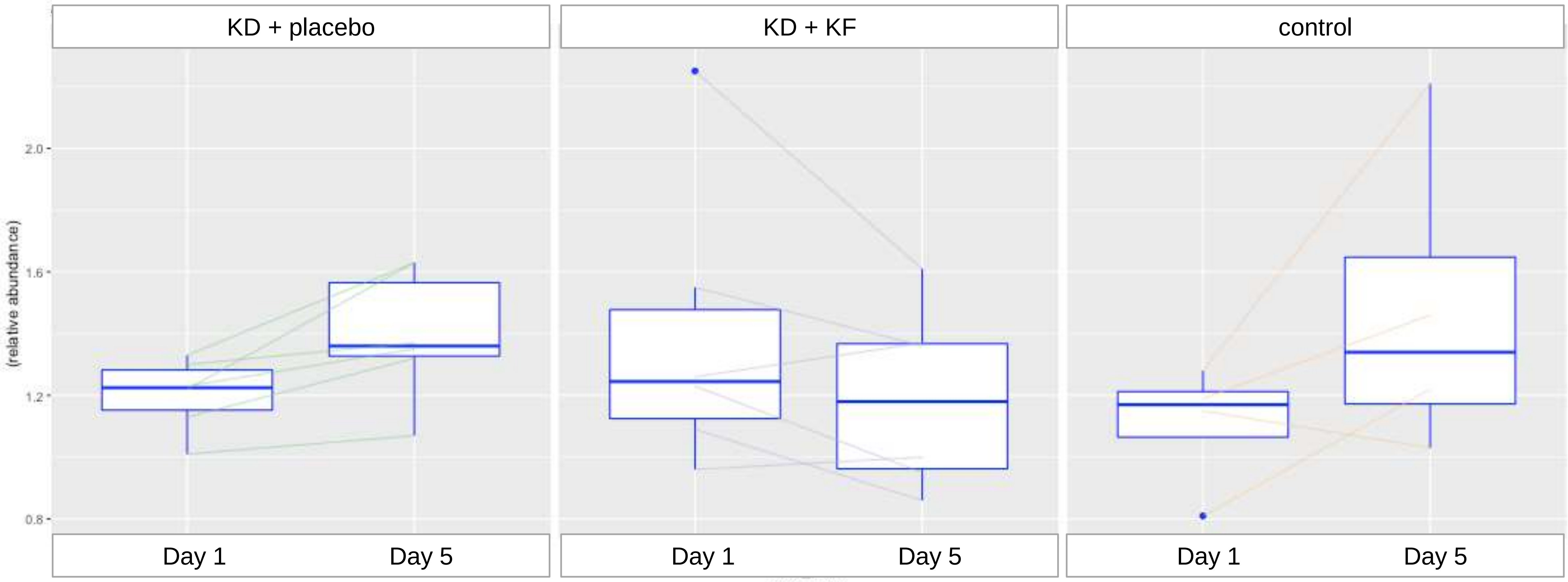

Supplement: Supplementary file 1 [file nutrients-14-01199-s001.zip › Supplemental_Figure_4.pdf]
